# The Association between Body Composition Measurements and Surgical Complications after Living Kidney Donation

**DOI:** 10.3390/jcm10010155

**Published:** 2021-01-05

**Authors:** Lisa B. Westenberg, Marco van Londen, Camilo G. Sotomayor, Cyril Moers, Robert C. Minnee, Stephan J. L. Bakker, Robert A. Pol

**Affiliations:** 1 Department of Surgery, University Medical Center Groningen, University of Groningen, 9713 GZ Groningen, The Netherlands; l.b.westenberg@umcg.nl (L.B.W.); c.moers@umcg.nl (C.M.); 2 Division of Nephrology, Department of Internal Medicine, University Medical Center Groningen, University of Groningen, 9713 GZ Groningen, The Netherlands; m.van.londen@umcg.nl (M.v.L.); c.g.sotomayor.campos@umcg.nl (C.G.S.); s.j.l.bakker@umcg.nl (S.J.L.B.); 3 Department of Surgery, Erasmus University Medical Center, Erasmus University Rotterdam, 3015 CN Rotterdam, The Netherlands; r.minnee@erasmusmc.nl

**Keywords:** living donation, nephrectomy, hand-assisted laparoscopic nephrectomy, body composition, complications

## Abstract

Obesity is considered a risk factor for peri- and postoperative complications. Little is known about this risk in overweight living kidney donors. The aim of this study was to assess if anthropometric body measures and/or surgical determinants are associated with an increased incidence of peri- and postoperative complications after nephrectomy. We included 776 living kidney donors who donated between 2008 and 2018 at the University Medical Center Groningen. Prenephrectomy measures of body composition were body mass index (BMI), body surface area (BSA), waist circumference, weight, and waist–hip ratio. Incidence and severity of peri- and postoperative complications were assessed using the Comprehensive Complication Index. Mean donor age was 53 ± 11 years; 382 (49%) were male, and mean BMI at donor screening was 26.2 ± 3.41 kg/m^2^. In total, 77 donors (10%) experienced peri- and postoperative complications following donor nephrectomy. Male sex was significantly associated with fewer surgical complications (OR 0.59, 0.37–0.96 95%CI, *p* = 0.03) in binomial logistic regression analyses. Older age (OR: 1.03, 1.01–1.05 95%CI, *p* = 0.02) and a longer duration of surgery (OR: 1.01, 1.00–1.01 95%CI, *p* = 0.02) were significantly associated with more surgical complications in binomial logistic regression analyses. Multinomial logistic regression analyses did not identify any prenephrectomy measure of body composition associated with a higher risk of surgical complications. This study shows that higher prenephrectomy BMI and other anthropometric measures of body composition are not significantly associated with peri- and postoperative complications following living donor nephrectomy.

## 1. Introduction

Transplantation of kidneys from living donors has many advantages in comparison with transplantation of deceased donor grafts. For patients with end-stage renal disease that undergo transplantation, patient and graft survival is better when transplanted with a graft from a living donor [1]. Although these findings support a need for more living kidney donors, the total number of living donor transplantations in the United States has remained constant since 2011 [2].

During screening for living kidney donation, body mass index (BMI) plays an important role in the assessment of a potential donor. A BMI ≥ 35 kg/m^2^ has been associated with an increased risk of peri- and postoperative complications such as surgical site infection, deep venous thrombosis development, and incisional hernia [3,4,5]. This risk of surgical complications was not significant in individuals with a BMI < 30 kg/m^2^ [6]. Therefore, many transplantation centres have decided that donors with a BMI ≥ 35 kg/m^2^ are not accepted for donation, and those who are obese with a BMI between 30 and 35 kg/m^2^ are advised to make lifestyle changes to reduce their weight [7,8]. Interestingly, studies on the effect of BMI as a risk factor for surgical complications in living donor nephrectomies report contradictory results [9,10], and especially, little is known about this risk in donors with a BMI between 30 and 35 kg/m^2^, who constitute a relatively large part of the living kidney donor population. Confronted with a changing living kidney donor pool due to the increasing prevalence of obesity worldwide [11] and the lack of a consensus on the threshold of BMI for living kidney donation acceptance criteria, the aim of this study is to assess whether BMI and other anthropometric body measures that are easily obtained in clinics are associated with an increased incidence of peri- and postoperative complications after nephrectomy.

## 2. Materials and Methods

### 2.1. Study Design

A total of 776 living kidney donors were included in this longitudinal prospective cohort study. Donor nephrectomies took place between 2008 and 2018 in the University Medical Center Groningen (The Netherlands). Potential donors were screened by a team of medical experts consisting of nephrologists, surgeons, radiologists, psychologists, and social workers. The main inclusion criterion was age >18 years of age at the time of donation. The exclusion criteria for donation were in accordance with the Dutch Guidelines for Evaluation of Potential Donors for Living Donor Kidney Transplantation from 2008 (i.e., BMI > 35 kg/m^2^, unable to provide informed consent, manifested Diabetes Mellitus, major cardiovascular risk factors, prior kidney disease or glomerular filtration rate (GFR) of <60 mL/min × 1.73 m^2^, monokidney, pregnancy, recent or active malignancies, chronic/active infection (e.g., HIV, HCV, HTLV, HBV), hypertension with end organ damage, inadequately regulated hypertension, proteinuria (>0.5 g/24 h), microscopical haematuria, and bilateral nephrolithiasis on CT scan) [9]. Informed consent was obtained from all participants.

Every donor underwent hand-assisted endoscopic donor nephrectomy, either laparoscopic (hand-assisted laparoscopy (HALN)) or retroperitoneoscopic (hand-assisted retroperitoneal nephrectomy (HARN)). Our hospital’s hand-assisted donor nephrectomy procedure has been described in detail in a previous publication [12]. All living kidney donors donated to recipients >18 years of age. The study was approved by the institutional ethical review board (METc 2014/077). All procedures were conducted in accordance with the Declarations of Helsinki.

### 2.2. Data Collection

Data were collected as part of the TransplantLines research project conducted at the UMCG [13]. During all visits, donors’ weight and height were measured by trained nurses. These measurements were used to calculate BMI (kg/m^2^) and body surface area (BSA); the latter was calculated using the Du Bois and Du Bois equation [14], a method most widely used in clinical practice. Waist and hip circumference were also measured at each visit of the donor as part of the TransplantLines study. Waist–hip ratio was calculated as the quotient of waist circumference and hip circumference.

Additional anthropometric, clinical, and laboratory measurements were extracted from the digital hospital registration system. Surgical complications were assessed using the Comprehensive Complication Index (CCI) [15], a continuous scale that measures surgical morbidity, considering all complications according to the Clavien–Dindo classification [16]. The CCI considers the incidence of each complication, using a specific calculation that results in a score between 0 and 100. Complications were prospectively recorded, and for this study, a full description of the reported complication was retrieved from the complication registry of the surgical department or otherwise extracted from the digital hospital registration system at our university hospital. As part of our follow-up protocol, donors regularly visit the hospital (i.e., 3 months, 1 year, 5 years, and 10 years after donation).

### 2.3. Statistical Analysis

Data were analysed using SPSS version 23.0 (IBM, Armonk, NY, USA). Categorical variables are presented as numbers with percentages and were analysed using the χ^2^ test or Fisher’s exact test. Normally distributed variables are presented as mean (standard deviation) and skewed variables are displayed as median [IQR], with analysis by means of Student’s *t*-test and the Mann–Whitney U test, respectively. We have performed these analyses for the total study population, complication vs. no complication, and BMI < 30 kg/m^2^ vs. BMI ≥ 30 kg/m^2^.

Since the Comprehensive Complication Index, a continuous score, was not distributed as a continuous variable in our study population, we performed logistic regression analyses. To determine which factors are associated with surgical complications, binomial and multinomial logistic regression analyses were performed. Each variable with a value of *p* < 0.05 with our outcome (i.e., a CCI score > 0) and variables known from the literature to be risk factors for perioperative complications were included in multinomial logistic regression models. Since living kidney donors need to be healthy individuals, most risk factors for perioperative complications (e.g., major comorbidities) do not apply. We have included the most important risk factors for perioperative complications pertaining to living kidney donors [17] in our analyses: longer duration of surgery has been associated with an increased risk of surgical complications such as surgical site infections, venous thromboembolism, and bleeding [18]. In addition, prior abdominal surgery shows strong evidence of association with an increased risk of intra-abdominal adhesions, complicating the procedure possibly leading to surgical complications [19]. Surgical technique could also affect the complication rate, since hand-assistance is associated with fewer surgical complications than an open laparoscopic procedure, and a retroperitoneoscopic approach might be associated with even fewer surgical complications [20].

Since BMI, the duration of surgery, and the occurrence of complications are interconnected, we have performed mediation analyses to investigate whether the duration of surgery might act as a mediator (Figures S1 and S2, Supplementary Materials). This analysis shows that there is an association between BMI and the duration of surgery, and between the duration of surgery and perioperative complications. However, since mediation requires the presence of a direct effect (in this case, an association between BMI and CCI > 0), which was not the case in our analyses, potential mediation of an association between BMI and CCI > 0 by the duration of surgery could not be assessed. Therefore, the duration of surgery could be included in our multinomial logistic regression models.

Independent variables in the multinomial logistic regression models were the body measures, age, sex, previous abdominal surgery, donor nephrectomy technique, and the duration of surgery. The dependent outcome variable was the category of CCI score. In all analyses, two-tailed values of *p* < 0.05 were seen as evidence for the presence of an association.

## 3. Results

We included 776 living kidney donors. Mean age at screening was 53 (SD: 11) years, and 49% were male. Mean BMI at donor screening was 26.2 (SD: 3.41) kg/m^2^ (Table 1). Mean waiting time between screening and donation was 9.7 months (SD: 12.8). The majority of donors donated their left kidney (*n* = 551, 72%), and the preferred surgical technique was hand-assisted laparoscopic donor nephrectomy in 679 (92%) donors. Conversion to another procedure was necessary in 17 (2%) cases (i.e., HARN to HALN in 13, HALN to open in 3, and HALN to HARN in 1). Mean duration of surgery was 215 (SD: 50) min, median blood loss was 50 [IQR: 50–150] mL, and median hospital length of stay was 4 [IQR: 4–5] days (Table 1).

The results of our subanalysis with a BMI < 30 kg/m^2^ vs. BMI ≥ 30 kg/m^2^ (Table S1, Supplement) show that donors with a BMI ≥ 30 kg/m^2^ had a higher measured GFR before donation (120.0 (SD: 24.0) vs. 111.5 (SD: 21.9) mL/min × 1.73 m^2^). Donors with a BMI ≥ 30 kg/m^2^ also had a longer duration of surgery (224.9 (SD: 50.1) vs. 213.0 (SD: 50.4) min) a and longer hospital length of stay (five [IQR: 4–5] vs. four [IQR: 4–5] days).

### 3.1. Surgical Determinants and Complications after Donor Nephrectomy

A total of 77 donors (10%) experienced peri- or postoperative complications following donor nephrectomy (Table 1). The most frequent complications were perioperative bleeding (19 donors, 22%), iatrogenic spleen lesion (13 donors, 15%), urinary retention (7 donors, 8%), or iatrogenic colon lesion (5 donors, 6%). The distribution of CCI scores for all complications is shown in Table 2. An overview of all complications is displayed in Supplementary Table S2. Forty-three donors experienced complications that required secondary surgical interventions. More female donors experienced complications compared to male donors (48 (12%) versus 29 (8%) male donors; *p* = 0.04). Donors that were older (mean: 57 (SD: 11) versus 54 (SD: 11) years; *p* = 0.02), those with a longer duration of surgery (228 (SD: 47) versus 213 (SD: 51) min; *p* = 0.02), those experiencing more intraoperative blood loss (125 [IQR: 50–463] versus 50 [IQR: 50–100] mL; *p* < 0.001), and those who had a longer hospital length of stay (five [IQR: 5–8] versus four [IQR: 4–5] days; *p* < 0.001) more frequently experienced a complication. All donors who underwent open donor nephrectomy experienced conversion from HALN to open due to a complication.

### 3.2. Determinants of Surgical Complications Following the Comprehensive Complication Index

Binomial logistic regression analyses with different body measures showed no significant association between BMI and surgical complications (OR for CCI > 0 vs. CCI = 0; 0.97, 0.91–1.04 95%CI, *p* = 0.43) (Table 3). Male gender was significantly associated with fewer surgical complications in binomial logistic regression analysis (OR: 0.59, 0.37–0.96 95%CI, *p* = 0.03). Older age (OR: 1.03, 1.01–1.05 95%CI, *p =* 0.02) and a longer duration of surgery (OR: 1.01, 1.00–1.01 95%CI, *p =* 0.02) were also associated with more surgical complications in binomial logistic regression analysis. In subanalyses among donors with a BMI between 30 and 35 kg/m^2^, we found no significant association with peri- and postoperative complications following nephrectomy (Table 3). Following multinomial logistic regression analysis with correction for possible confounders, no measure for body composition was a significant determinant of surgical complications (Table 4).

## 4. Discussion

This study showed that in our cohort of living donors, there was no significant association between BMI or other anthropometric body measures and peri- and postoperative complications.

Higher BMI has previously been associated with an increased risk of peri- and postoperative complications in different study populations. Incidence of surgical site infection increases with increasing BMI in general surgery patients [3], possibly due to low regional perfusion and oxygen tension resulting from excessive subcutaneous fat tissue impairing wound healing. Duration of surgery is also often prolonged in obese individuals [21], adding to the risk of surgical site infection [22]. Obese individuals were at increased risk of major postoperative complications following surgery for gastrointestinal malignancy and renal cancer [23,24]. Due to a larger BSA and more complex fluid management, risk of intraoperative hypothermia is increased in obese individuals, predisposing them to surgical and thromboembolic complications [5,23]. In overweight and obese donors, a larger extraction incision is usually necessary due to the thicker layer of adipose tissue, leading to a higher risk for abdominal wall complications (e.g., incisional hernia and wound infections) [6,25].

Different from the aforementioned studies, our results, from one of the largest prospective cohorts, suggest no significant deleterious effect of high fat mass on peri- and postoperative complications following donation. A possible explanation might be that living kidney donors differ significantly from other surgical populations in which they have little to no comorbidities at the time of surgery [18]. Although the donor population in our study did not allow an analysis of donors with a BMI ≥ 35 kg/m^2^, we found no evidence of an association between BMI and surgical complications following donation in subanalyses of obese donors with a BMI between 30 and 35 kg/m^2^.

Whether BMI is the best way to measure obesity remains unclear. Various other anthropometric measures, such as BSA, waist circumference, and waist–hip ratio, are also frequently used in clinical settings but with varying results with respect to each other. Cross-sectional surveys evaluating the predictive power of BMI and waist circumference have shown waist circumference to be a better predictor for obesity-related comorbidities than solely BMI [26,27]. In assessing obesity-related renal effects, waist–hip ratio appears to be superior to both BMI and waist circumference [28].

We detected strong evidence of an association between longer duration of surgery and peri- and postoperative complications following donor nephrectomy, which are, of course, interconnected. Although the occurrence of a surgical complication might result in a prolonged duration of surgery, the likelihood of surgical complications such as surgical site infections, venous thromboembolism, bleeding, hematoma formation, and necrosis also increases with prolonged duration of surgery [29]. A recent systematic review and meta-analysis demonstrated that the risk of complications approximately doubled with prolonged operative duration and the risk of surgical complications increased by 14% for every 30 min of additional operating time [29]. Although the underlying mechanisms are not yet fully understood, a prolonged microbial exposure [30] and a diminishing efficacy of antimicrobial prophylaxis over time [31] appear to be contributing factors. Venous thromboembolism formation is more likely to occur with prolonged surgical procedures due to an increased risk of blood stasis, coagulation activation, and endothelial damage, also known as Virchow’s triad [32]. Obesity prolongs the duration of surgery and can therefore also lead to a higher risk of aforementioned complications [22].

Living kidney donors are a unique group of surgical patients, given that a low comorbidity burden is required to be eligible for donation. In current donor screening guidelines, BMI is a widely applied measure for assessing obesity [9]. It is, however, a poor estimate of fat mass distribution. Muscular individuals or those with more subcutaneous fat can have a similar BMI to individuals with more visceral fat, but these different types of high BMI are associated with different disease risks [33]. Contradictory to what is generally known about obesity and its effect on disease and mortality risk, some studies show a protective effect of high BMI in patients [34]. This apparent protective effect is often referred to as the “obesity paradox” and also underlines that BMI poorly reflects the actual balance or imbalance in fat mass distribution and muscle volume. Therefore, we need to incorporate more reliable tools to measure body composition when defining obesity and determining its effect on postsurgical outcome. Bioelectrical impedance analysis (BIA) is a tool for assessing body composition by measuring the resistance of the body as a conductor to a very small alternating electrical current. This technique might provide a more detailed and reliable analysis of fat and muscle mass, enabling assessment of the association between these two determinants of body composition and peri- and postoperative complications after surgery. This method, however, is not yet sufficiently validated among living kidney donors. Another promising technique to assess the risk of surgical complications following donor nephrectomy is a volumetric measurement of perirenal fat mass based on CT-scans, which shows a stronger correlation with outcome measures of laparoscopic donor nephrectomy than BMI alone [35]. Future studies, also by our group, should investigate other parameters defining the outcome of donation based on body composition and BMI, such as slow or delayed graft function, long-term renal function, and development of comorbidity. There is also a call for more studies assessing obesity in living kidney donors with a variety of ethnicities, especially since donors with an African background seem to more commonly be obese and develop conditions such as chronic kidney disease, proteinuria, and nephrotic syndrome [36,37]. In addition, data on lifetime risk of chronic kidney disease and mortality in young living kidney donors are sparse and should be a focus of future studies.

Our study has a few limitations that need to be addressed. Although our study consists of a large cohort of living kidney donors and missing data for predonation body measures was limited in this study, the exclusion criteria that were applied in donor screening [9] (e.g., BMI > 35 kg/m^2^, manifested Diabetes Mellitus, major cardiovascular risk factors, proteinuria > 0.5 g/24 h) affected our results. Especially the exclusion of potential donors with a BMI > 35 kg/m^2^ resulted in a narrow range of BMI, making our study population a selection of the total group of living kidney donors, which might not be representative to other kidney donor populations where this criterion is not applied in donor screening guidelines. The incidence of complications in the subgroup with a BMI of 30–34.99 was low. Future studies with the inclusion of a larger number of donors with a BMI in this range are required to further investigate the effect of living kidney donation on the development of complications in this group. Although our study shows a similar complication rate to observations in the United States [22], complications might have been underreported, especially following procedures performed in the early years of our data collection, which might have influenced the results. Furthermore, living kidney donors are part of a highly selected population. Therefore, our results cannot automatically be extrapolated to other kinds of surgery and to other populations. We have used literature on living kidney donors when available but referred to other kinds of surgery or other study populations when this was lacking.

In conclusion, this study shows no strong evidence of an association between BMI and other anthropometric body measures and peri- and postoperative complications following donor nephrectomy and should therefore be no reason to refrain from surgery.

## Figures and Tables

**Table 1:** Characteristics of the study population.

	Study Population	Donors without Complication	Donors with Complication	*p*
	*n* = 776	*n* = 699	*n* = 77	
Gender, *n* (%)				0.04
Male	382 (49.2%)	353 (50.5%)	29 (37.7%)	
Age at nephrectomy, years	54 ± 11	53.7 ± 11	56.8 ± 11	0.02
Weight, kg	80.5 ± 13.2	80.8 ± 13.3	78.2 ± 12.6	0.11
Length, cm	175.1 ± 9.6	175.3 ± 9.5	173.7 ± 10.6	0.15
BMI, kg/m^2^	26.2 ± 3.41	26.2 ± 3.4	25.9 ± 3.4	0.43
BSA, m^2^	1.96 ± 0.20	1.96 ± 0.19	1.92 ± 0.20	0.1
Hip size, cm	98.9 ± 7.6	98.9 ± 7.6	98.8 ± 7.4	0.93
Waist size, cm	91.1 ± 10.5	91.2 ± 10.6	91.0 ± 10.2	0.87
Waist–hip ratio	0.92 ± 0.11	0.92 ± 0.11	0.92 ± 0.09	0.84
Blood pressure				
Systolic, mmHg	126.4 ± 12.8	126.4 ± 12.9	126.6 ± 12.3	0.91
Diastolic, mmHg	75.7 ± 8.99	75.7 ± 9.0	75.9 ± 9.2	0.89
mGFR, mL/min × 1.73 m^2^	112.8 ± 22.4	113.1 ± 22.7	109.6 ± 19.4	0.19
Side nephrectomy				0.99
Left, *n* (%)	551 (72%)	495 (71.9%)	56 (72.7%)	
Right, *n* (%)	214 (28%)	193 (28.1%)	21 (27.3%)	
Previous abdominal surgery, *n* (%)	26 (3.4%)	25 (3.6%)	1 (1.3%)	0.47
Surgical technique				
HALN, *n* (%)	679 (92.3%)	605 (91.8%)	74 (96.1%)	0.27
HARN, *n* (%)	55 (7.5%)	54 (8.2%)	1 (1.3%)	0.05
Open, *n* (%)	2 (0.3%)	0 (0%)	2 (2.6%)	0.003
Duration of surgery, min	215 ± 50	213 ± 51	228 ± 47	0.02
Blood loss, mL	50.0 (50.0–150.0)	50.0 (50.0–100.0)	125.0 (50.0–462.5)	<0.001
HLOS, days	4.0 (4.0–5.0)	4.0 (4.0–5.0)	5.0 (5.0–8.0)	<0.001
Conversion rate, *n* (%)				
No, primary HALN	666 (90.5%)	593 (90.0%)	73 (94.8%)	0.25
No, primary	53 (7.2%)	52 (7.9%)	1 (1.3%)	0.06
HARN				
No, primary open	0 (0%)	0 (0%)	0 (0%)	
Conversion HARN	13 (1.8%)	12 (1.8%)	1 (1.3%)	1
to HALN				
Conversion HARN	0 (0%)	0 (0%)	0 (0%)	
to open				
Conversion HALN	3 (0.4%)	1 (0.2%)	2 (2.6%)	0.03
to open				
Conversion HALN	1 (0.1%)	1 (0.2%)	0 (0%)	1
to HARN				

Values of variables are given as mean ± standard deviation, median [interquartile range], or *n* (%); BMI, body mass index (kg/m^2^); BSA, body surface area (m^2^); mGFR, measured glomerular filtration rate (mL/min × 1.73 m^2^); HALN, hand-assisted laparoscopy; HARN, hand-assisted retroperitoneal nephrectomy; HLOS, hospital length of stay.

**Table 2:** Distribution of Comprehensive Complication Index scores within the study population.

CCI Score	Number of Donors *n* (%)
0	699 (90)
8.7	20 (3)
12.2	1 (0.1)
20.9	11(1.4)
22.6	1 (0.1)
29.6	1 (0.1)
33.7	41 (5)
35.9	1 (0.1)
44.9	1 (0.1)

**Table 3:** Binomial logistic regression analysis of the association of anthropometrics with surgical complications (Comprehensive Complication Index score).

	Odds Ratio for CCI > 0 vs. CCI = 0		
	OR	95% CI	*p*-Value
Gender			
Female	1	-	-
Male	0.59	0.37–0.96	0.03
Age, years	1.03	1.01–1.05	0.02
Previous abdominal surgery			
No	1	-	-
Yes	2.82	0.38–21.1	0.31
Surgical technique			
HALN	1	-	-
HARN	6.61	0.90–48.4	0.06
Duration surgery, min	1.01	1.00–1.01	0.02
Weight, kg	0.99	0.97–1.00	0.11
BMI, kg/m^2^	0.97	0.91–1.04	0.43
BMI 30–34.99 kg/m^2 a^	1.32	0.64–2.73	0.45
BSA, m^2^	0.35	0.10–1.21	0.1
Waist circumference, cm	1	0.97–1.02	0.87
Waist–hip ratio	0.78	0.07–8.91	0.84

The reference category is CCI = 0 (i.e., no complication). BMI, body mass index (kg/m^2^); BSA, body surface area (m^2^); HALN, hand-assisted laparoscopy; HARN, hand-assisted retroperitoneal nephrectomy. a. A total of nine (8%) of donors with a BMI of 30–34.99 kg/m^2^ experienced one or more surgical complications.

**Table 4:** Multinomial logistic regression analysis of the association of anthropometrics with surgical complications (with Comprehensive Complication Index score classified into categories).

	CCI 0.1–20.0		CCI 20.1–30.0		CCI > 30.0	
	*n* = 21		*n* = 13		*n* = 43	
	OR (95%CI)	*p*-Value	OR (95%CI)	*p*-Value	OR (95%CI)	*p*-Value
Weight, kg						
Model 1	0.99 (0.95–1.03)	0.59	1.01 (0.97–1.06)	0.61	0.99 (0.96–1.02)	0.56
Model 2	0.99 (0.95–1.03)	0.51	1.02 (0.97–1.07)	0.46	0.98 (0.95–1.01)	0.27
BMI, kg/m^2^						
Model 1	0.92 (0.81–1.06)	0.24	0.93 (0.79–1.10)	0.42	1.00 (0.92–1.10)	0.92
Model 2	0.91 (0.79–1.04)	0.18	0.96 (0.82–1.14)	0.66	0.96 (0.86–1.06)	0.39
BSA, m^2^						
Model 1	0.77 (0.04–15.4)	0.87	13.4 (0.36–490.0)	0.16	0.40 (0.05–3.44)	0.4
Model 2	0.71 (0.04–14.2)	0.82	14.2 (0.35–566.9)	0.16	0.31 (0.03–3.46)	0.34
Waist circumference, cm						
Model 1	1.00 (0.95–1.05)	0.89	0.99 (0.94–1.06)	0.82	1.01 (0.98–1.04)	0.57
Model 2	0.99 (0.95–1.04)	0.74	1.01 (0.95–1.07)	0.69	0.99 (0.96–1.03)	0.72
Waist–hip ratio						
Model 1	1.38 (0.01–188.6)	0.9	0.03 (0.00–58.5)	0.37	3.73 (0.18–76.3)	0.39
Model 2	0.88 (0.01–119.9)	0.96	0.36 (0.00–324.1)	0.77	1.98 (0.05–75.5)	0.71

The Comprehensive Complication Index (CCI) [15] score is classified into categories (CCI 0.1–20.0; CCI 20.1–30.0; CCI > 30.0) and compared to the reference category of CCI = 0 (e.g., no complication). BMI, body mass index (kg/m^2^); BSA, body surface area (m^2^). Model 1 is age- and sex-adjusted. Model 2 is adjusted for age, sex, previous abdominal surgery, donor nephrectomy technique, and duration of surgery.

## Data Availability

The data presented in this study are available on request from the corresponding author. The data are not publicly available due to privacy restrictions.

## Supplementary Materials

The following are available online at https://www.mdpi.com/2077-0383/10/1/155/s1, Figure S1: Standardized Regression Coefficients and Odds Ratios for the Relationship Between Body Mass Index and Comprehensive Complications Index score above zero as Mediated by Duration of Surgery, Figure S2: Standardized Regression Coefficients and Odds Ratios for the Relationship Between Duration of Surgery and Comprehensive Complications Index score above zero as Mediated by Body Mass Index, Table S1: Characteristics of the study population by BMI category, Table S2: Overview of complications.
