# Supplementary material for: The Association between Body Composition Measurements and Surgical Complications after Living Kidney Donation
Source: J Clin Med. 2021 Jan 5;10(1):155. doi: 10.3390/jcm10010155 (PMC7794883; doi:10.3390/jcm10010155)
Supplement: Supplementary file 1 [file jcm-10-00155-s001.pdf]

Table S1. Characteristics of the study population by BMI category.

	**BMI <30 kg/m^2 n=658**	**BMI ≥30 kg/m^2 n=114**	***p***
**Gender, n (%)**			0.88
**Male**	326 (49.5)	55 (48.2)	
**Age at nephrectomy, years**	54 ± 11.0	55 ± 10.2	0.35
**Blood pressure**			
**Systolic, mmHg**	126.0 ± 12.5	128.7 ± 14.3	0.07
**Diastolic, mmHg**	75.6 ± 8.88	76.6 ± 9.58	0.27
**mGFR, mL/min*1.73m^2**	111.5 ± 21.9	120.0 ± 24,0	0.**001**
**Side nephrectomy**			0.85
**Left, n (%)**	466 (71.8)	82 (73.2)	
**Right, n (%)**	183 (28.2)	30 (26.8)	
**Previous abdominal surgery, n (%)**	23 (3.5)	3 (2.6)	0.85
**Surgical technique**			
**HALN, n (%)**	570 (91.3)	105 (97.2)	0.06
**HARN, n (%)**	52 (8.3)	3 (2.8)	0.07
**Open, n (%)**	2 (0.3)	0 (0)	1.00
**Duration of surgery, min.**	213.0 ± 50.4	224.9 ± 50.1	0.**03**
**Blood loss, mL**	50 [50-150]	50 [50-200]	0.76
**HLOS, days**	4 [4-5]	5 [4-5]	0.**05**
**Conversion rate, n (%)**			
**No, primary HALN**	558 (89.4)	104 (96.3)	0.**04**
**No, primary HARN**	50 (8.0)	3 (2.8)	0.08
**No, primary open**	-	-	
**Conversion HARN to HALN**	12 (1.9)	1 (0.9)	0.74
**Conversion HARN to open**	-	-	
**Conversion HALN to open**	3 (0.5)	0 (0)	1.00
**Conversion HALN to HARN**	1 (0.2)	0 (0)	1.00

Values of variables are given as mean ± standard deviation, median [interquartile range], or n (%)

BMI, Body Mass Index (kg/m2); BSA, Body Surface Area (m2); mGFR, measured Glomerular Filtration Rate (mL/min*1.73m2); HALN, Hand-Assisted Laparoscopy; HARN, Hand-Assisted Retroperitoneal Nephrectomy; HLOS, Hospital Length of Stay.

Table S2. Overview of complications.

Complication	Incidence n (%)
Perioperative bleeding	19 (22)
Iatrogenic spleen lesion	13 (15)
Urinary retention	7 (8)
Iatrogenic colon lesion	5 (6)
Postoperative bleeding (hematoma)	4 (5)
Pneumonia treated with antibiotics	4 (5)
Hypokalemia	4 (5)
Postoperative pain treated with analgesics	3 (4)
Fecal retention	2 (2)
Nausea without intervention	1 (1)
Nausea with intervention	1 (1)
Abscess	1 (1)
Postoperative pancreas leakage	1 (1)
Chyle leakage	1 (1)
Fever and irritated peritoneum	1 (1)
Foreign object left in patient	1 (1)
Wound infection	1 (1)
Perioperative hypotension	1 (1)
Post-operative vasovagal collapse	1 (1)
VRE infection	1 (1)
Postoperative omental necrosis	1 (1)
Chest pain	1 (1)
Lung embolus	1 (1)
Urinary tract infection	1 (1)
Prolonged treatment with drain	1 (1)
Wound leakage	1 (1)
Iatrogenic bladder lesion	1 (1)
Iatrogenic pancreas lesion	1 (1)
Atelectasis	1 (1)
Pneumothorax	1 (1)
Renal insufficiency	1 (1)
Perioperative hypothermia	1 (1)
Indicated relaparoscopy	1 (1)

Figure S1. Standardized Regression Coefficients and Odds Ratios for the Relationship Between Body Mass Index and Comprehensive Complications Index score above zero as Mediated by Duration of Surgery.

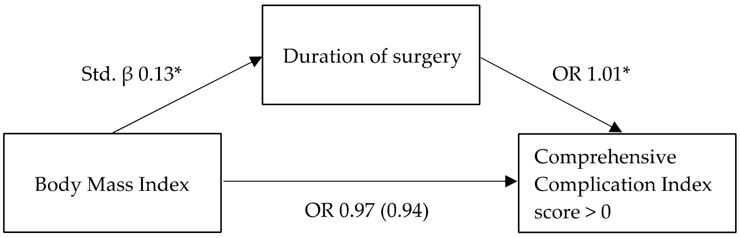

* $p < 0.05$

Figure S2. Standardized Regression Coefficients and Odds Ratios for the Relationship Between Duration of Surgery and Comprehensive Complications Index score above zero as Mediated by Body Mass Index.

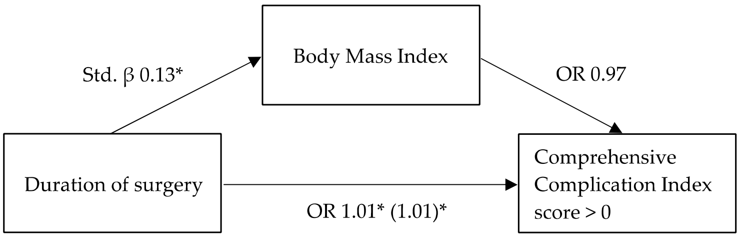

* $p < 0.05$
